# The biology of DHX9 and its potential as a therapeutic target

**DOI:** 10.18632/oncotarget.8446

**Published:** 2016-03-28

**Authors:** Teresa Lee, Jerry Pelletier

**Affiliations:** ^1^ Department of Biochemistry, McGill University, Montreal, Quebec, Canada; ^2^ Department of Oncology, McGill University, Montreal, Quebec, Canada; ^3^ Department of Rosalind and Morris Goodman Cancer Research Center, McGill University, Montreal, Quebec, Canada

**Keywords:** DHX9, helicase, RNA helicase A, DExD/H-box, Maleless

## Abstract

DHX9 is member of the DExD/H-box family of helicases with a “DEIH” sequence at its eponymous DExH-box motif. Initially purified from human and bovine cells and identified as a homologue of the *Drosophila* Maleless (MLE) protein, it is an NTP-dependent helicase consisting of a conserved helicase core domain, two double-stranded RNA-binding domains at the N-terminus, and a nuclear transport domain and a single-stranded DNA-binding RGG-box at the C-terminus. With an ability to unwind DNA and RNA duplexes, as well as more complex nucleic acid structures, DHX9 appears to play a central role in many cellular processes. Its functions include regulation of DNA replication, transcription, translation, microRNA biogenesis, RNA processing and transport, and maintenance of genomic stability. Because of its central role in gene regulation and RNA metabolism, there are growing implications for DHX9 in human diseases and their treatment. This review will provide an overview of the structure, biochemistry, and biology of DHX9, its role in cancer and other human diseases, and the possibility of targeting DHX9 in chemotherapy.

## INTRODUCTION TO DHX9 AND DEXD/H-BOX HELICASES

DHX9 (also known as Nuclear DNA Helicase II (NDH II) and RNA Helicase A (RHA)) is an NTP-dependent helicase protein capable of unwinding both RNA and DNA [1], as well as aberrant polynucleotide structures [2]. It is a member of the DExH-box family of helicases, so-named for the conserved Asp-Glu-Ile-His (DEIH) sequence in its helicase core domain, and is part of the larger superfamily (SF) 2 category of helicases, which comprise both DExH and DExD helicases. DHX9 is a multi-domain, multi-functional protein, with regulatory roles in DNA replication, transcription, translation, RNA processing and transport, microRNA processing, and maintenance of genomic stability. Homologues have been characterized in human, bovine, mouse, *Drosophila*, *C. elegans*, and *Arabidopsis*, although the majority of the research has focused on human DHX9. Many of the biological processes in which DHX9 participates are deregulated during oncogenesis, or are hijacked by viruses to promote their own replication. A wealth of recent studies have implicated DHX9 in human diseases such as various cancers and viral infections, and there is evidence supporting the targeting of DHX9 in disease intervention. This review will discuss the structural, biochemical and biological properties of DHX9 as well as its implications in disease.

**Figure 1:**
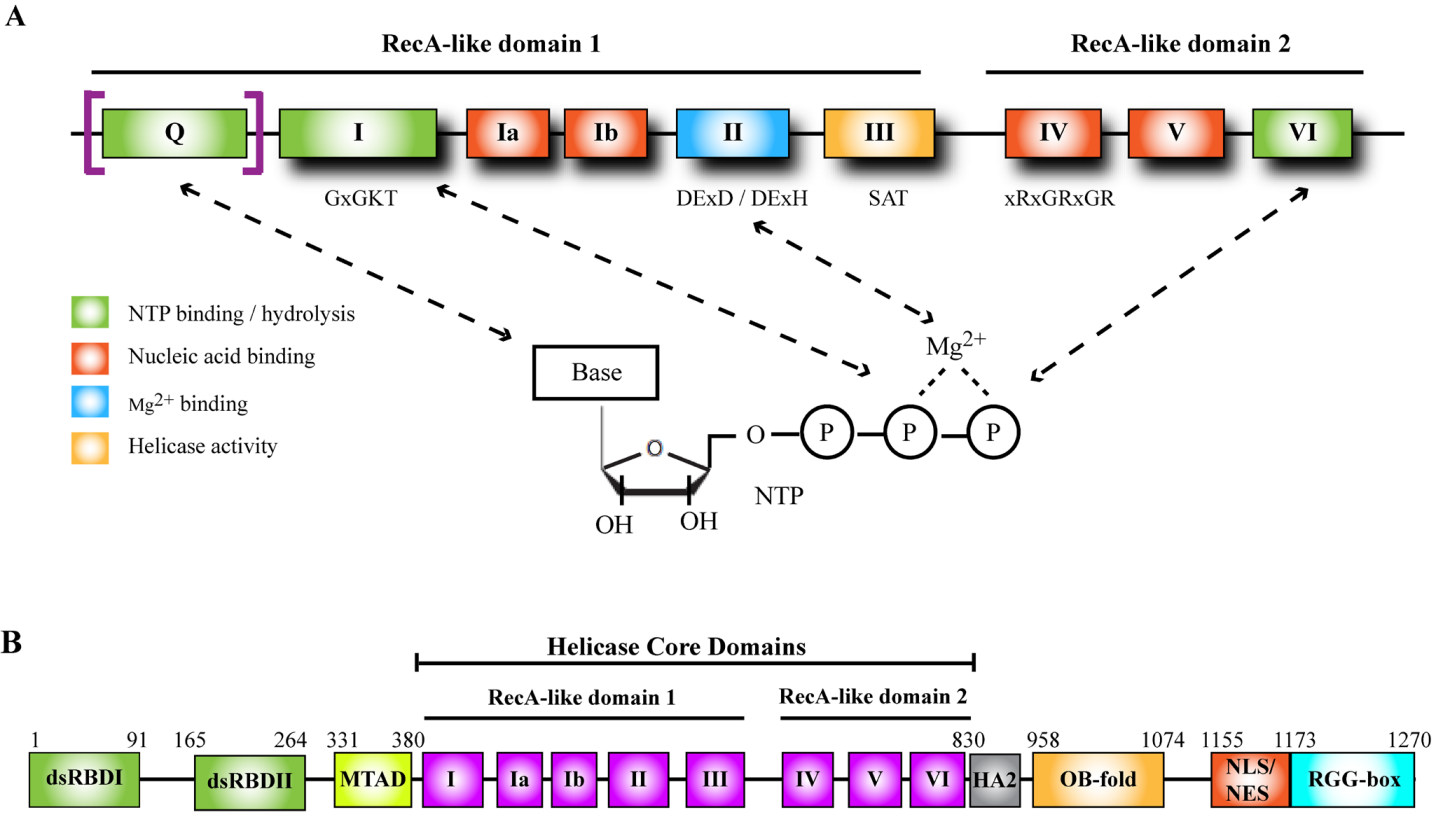
Helicase domain of DExD/H-box helicases and functional domains in DHX9 **A.** Conserved sequence motifs in the helicase core domain of DDX and DHX helicases. Consensus sequences are shown below some of the motifs. “x” represents any amino acid. The Q-motif is present in DDX but not DHX box helicases (including DHX9) and confers specificity for ATP binding. **B.** Schematic representation of DHX9 functional domains. Numbers indicate amino acid positions in human DHX9. dsRBD, double-stranded RNA binding domain; MTAD, minimal transactivation domain; HA2, helicase-associated domain 2; OB-fold, oligonucleotide/oligosaccharide-binding fold; NLS, nuclear localization signal; NES, nuclear export signal.

Helicases are enzymes which catalyze the energy-dependent remodeling of nucleic acids. They utilize the free energy of binding and hydrolysis of nucleotide triphosphates to unwind nucleic acid duplexes or dissociate ribonucleoprotein complexes [3, 4]. Helicases are categorized into six superfamilies (SF1 - SF6) according to their amino acid sequence and structure [3, 5]. SF1 and SF2 helicases are structurally similar, consisting of two globular RecA-like domains in their core helicase region, each comprised of 5 β-strands surrounded by 5 α-helices, and act as monomers or dimers. SF3-SF6 family members contain only one RecA-like domain and form hexameric rings [3, 5]. Members of the largest superfamily, SF2, contain a signature helicase domain consisting of 7-9 evolutionarily conserved motifs, and are further subdivided into 2 main subfamilies based on the consensus sequence in motif II, the major site of NTP-binding and hydrolysis: the DExD-box (DDX) family, which contains the conserved sequence Asp-Glu-x-Asp (where x is any amino acid), and the DExH-box (DHX) family, which is defined by the sequence Asp-Glu-x-His [6]. The DExD/H box interacts with the β and γ phosphates of the NTP *via* Mg^2+^ (Figure 1A). Structural and biochemical evidence suggests that in addition to motif II, motifs I, VI, and the Q-motif also participate in NTP-binding and hydrolysis. Motif III is responsible for coupling NTP hydrolysis to nucleic acid unwinding, and motifs Ia, Ib, IV, and V are involved in nucleic acid binding (Figure 1A) [7]. Despite their overall structural similarities, DDX and DHX family members exhibit significant differences in key residues within the conserved motifs. DHX helicases are also distinct from DDX members in that the former can hydrolyze different NTPs whereas the latter is generally specific for ATP. This is attributed to the Q-motif, which is present in DDX but not DHX helicases, and makes contact with the adenine base [6–8]. The helicase motifs are organized into two RecA-like domains and NTP-binding occurs at the cleft between the two domains [3].

Helicases play a role in a wide variety of cellular processes. Different family members possess the ability to unwind duplexes of DNA or RNA, as well as heteroduplexes and more complex polynucleotide structures (e.g. triple-stranded DNA). Other helicases may act as RNA clamps or chaperones to aid in RNA folding. These functions have made them important players in nucleic acid unwinding and remodeling during replication, transcription, translation, DNA repair, RNA splicing and editing, ribosome biogenesis, RNA transport, and RNA decay [7, 8]. They have been implicated in normal development and the cellular antiviral response, and helicase defects have been associated with genetic disorders and cancers [9–15].

## BIOCHEMICAL AND STRUCTURAL CHARACTERIZATION OF DHX9

### Discovery of DHX9

Mammalian DHX9 was first purified from the nuclear fraction of calf thymus and designated Nuclear DNA Helicase II (NDHII) [16]. The human homologue, known as RNA helicase A (RHA), was isolated shortly afterwards from nuclear extracts of HeLa cells [17]. Initial helicase assays demonstrated double-stranded DNA unwinding activity in bovine DHX9 and RNA unwinding activity with the human orthologue; however, subsequent studies showed that DHX9 from both species could unwind both DNA and RNA in an NTP-dependent manner [1]. cDNA clones for bovine and human DHX9 were obtained by immunoscreening cDNA libraries, and molecular cloning revealed that they were homologous to the *Drosophila* melanogaster protein Maleless (MLE) [16, 18]. MLE was first discovered in *Drosophila* as a gene which when mutated caused lethality in male zygotes, and was found to play a role in X-chromosome dosage compensation in males [19, 20]. Subsequent work characterized DHX9 homologues in mouse [21], *C. elegans* [22], and Arabidopsis [23].

### DHX9 functional domains and structure

**Figure 2:**
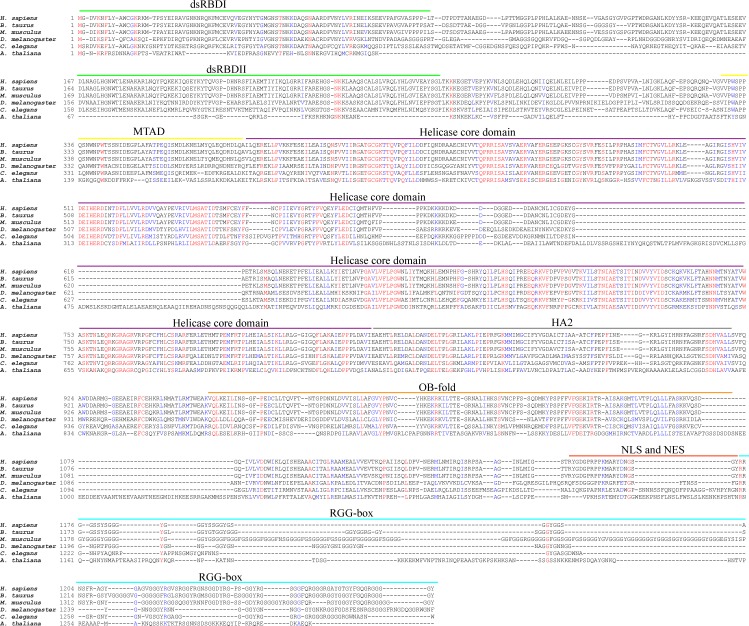
Conservation of DHX9 across various species Multiple sequence alignment of DHX9 homologues from human (*H. sapiens*) (NCBI accession # NP_001348), bovine (*B. taurus*) (NP_776461), mouse (*M. musculus*) (NP_031868), *Drosophila* (*D. melanogaster*) (NP_476641), *C. elegans* (NP_495890), and *Arabidopsis* (*A. thaliana*) (NP_850154). Red text indicates residues that are identical in all species. Blue text indicates residues with high similarity amongst species. The sequence alignment was generated using T-Coffee and visualized with BoxShade.

The human DHX9 gene maps to the prostate cancer susceptibility locus on chromosome 1q25, with a pseudogene, DHX9P, mapping to chromosome 13q22 [24]. The active gene is comprised of 29 exons and encodes a 1270 amino acid, 140-kDa protein [25]. In the mouse, DHX9 maps to chromosome 1 [21]. In common with other DHX family members, sequence analysis revealed that DHX9 contains a helicase core domain consisting of 8 motifs (Figure 1B). As with other SF2 helicases, the core region is subdivided into two RecA-like domains, with motifs I-III residing in domain 1 and motifs IV-VI in domain 2. In addition to the helicase core domain (which spans amino acid (aa) residues 380 to 830 in humans), DHX9 contains 2 double stranded RNA-binding domains (dsRBDs) at its N-terminus [25]. The minimal transactivation domain (MTAD), the site of RNA polymerase II (PolII) interaction (see Biological Functions of DHX9, Transcriptional regulation), is situated between dsRBDII and motif I of the helicase core domain. A helicase-associated domain 2 (HA2) is present adjacent to the C-terminal end of the helicase core domain [26]. At the C-terminus of DHX9 lies an oligonucleotide/oligosaccharide-binding fold (OB-fold) [27], overlapping nuclear localization/export signals [28], and a glycine-rich RGG-box capable of binding single-stranded nucleic acids [25] (Figures 1B and 2).

Sequence alignment showed a high degree of homology amongst DHX9 from various species. Human DHX9 exhibits 93% identity to the bovine homologue, 90% identity to the murine homologue, and 50%, 42%, and 27% identity to the *D. melanogaster, C. elegans*, and *Arabidopsis* homologues respectively (Figure 2). The helicase core region is highly conserved amongst all species, whereas the N- and C-terminal regions exhibit more variation. Notably, the RGG-box at the C-terminus of murine DHX9 is significantly more extended than that of DHX9 in other species [29] (Figure 2).

The structure of DHX9 has been partially solved. X-ray crystallography was used to determine the structure of domain 1 of human DHX9's helicase core in the presence of ADP and Mn^2+^. This region spans residues 325 to 563 and includes motifs I to III as well as the 90 amino-acid region immediately upstream of motif I, the first 30 residues of which form part of the MTAD [30]. Motifs I-III form a RecA-like α/β core consisting of 5 parallel β-strands alternating with 5 α-helices, a structure which is conserved amongst DExD/H helicases. The conserved motifs are in close proximity to each other, allowing them to cooperatively form nucleotide and nucleic acid binding sites. Binding to the NTP is accomplished *via* stacking interactions between the base of the nucleotide and an arginine residue in motif Ia, and interactions between the phosphates and motif I. The aspartic and glutamic acid residues of the DEIH-box binds to the divalent cation, which serves to further coordinate the NTP. The structural data shows that the Q-motif, which confers nucleotide specificity (to ATP) in DExD helicases, is not present in DHX9 and that DHX9 lacks base-selective contacts, thereby enabling it to utilize all four NTPS (ATP, GTP, CTP, and UTP) for its energy requirements. The region immediately preceding motifs I-III consists of 3 α-helices arranged perpendicular to each other on the surface of the α/β core, while the MTAD region consists of 2 short β-strands which lie in a hydrophobic groove on the surface of the helicase core [30].

The structure of the two dsRBD domains has also been elucidated. Both dsRBDI and dsRBDII are arranged into a core α-β-β-β-α fold, with the two α-helices lying on one surface of the three-stranded antiparallel β-sheets, and both domains were able to co-crystallize with dsRNA. Interaction with dsRNA is mediated primarily by surface-exposed residues which tend to be highly conserved amongst dsRBDs of various proteins [31]. Despite the structural similarities, dsRBDI and dsRBDII only share 21% sequence similarity, a fact that may confer selectivity in terms of unique RNA or protein interactions. The structures of dsRBDI and dsRBDII in murine DHX9 have been determined by NMR spectroscopy and shown to be very similar to their human counterparts [32, 33].

### *In vitro* characterization of DHX9

**Figure 3:**
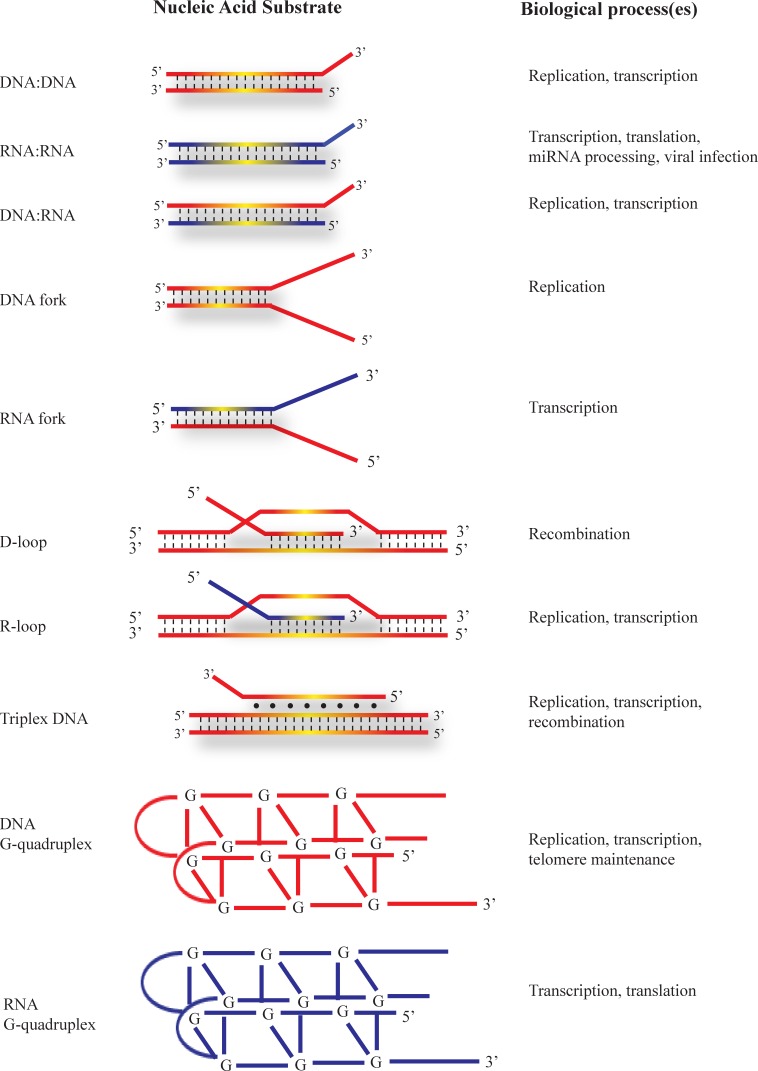
Nucleic acid substrates unwound by DHX9 Schematic representation of nucleic acid substrates that can be remodeled by DHX9. DNA strands are coloured in red and RNA strands in blue. The biological processes in which the substrates occur are indicated. Substrates are arranged from top to bottom in approximate order of increasing complexity. See text for a detailed description.

*In vitro* studies using human, bovine, and *Drosophila* DHX9 demonstrated that all three homologues are able to bind both DNA and RNA [1, 17, 34]. DHX9 unwinds double stranded (ds) DNA and RNA, as well as DNA/RNA hybrids, with RNA-containing duplexes being unwound more efficiently than dsDNA [35]. It exhibits a preference for substrates with a short single-stranded non-complementary 3′ tail - a feature which is commonly found at replication forks (Figure 3). DHX9 translocates in the 3′ to 5′ direction and is able to utilize all dNTPs and rNTPs for its unwinding activity, with similar K_M_ values, in concordance with the lack of NTP selectivity (due to the absent Q-motif) demonstrated by structural data [1, 17, 30, 34, 36]. *In vitro* experiments have shown that human DHX9 also unwinds DNA or RNA forks composed of either partially complementary DNA duplexes or DNA/RNA hybrids respectively [37] (Figure 3). Related to fork assembly are DNA displacement loops (D-loops), which are formed when single-stranded DNA “invades” a complementary duplex, displacing one strand while forming Watson-Crick base pairs with the complementary strand, and which are found during initiation of homologous recombination [38–40]. Similarly, RNA displacement loops (R-loops) are created when single-stranded RNA invades a DNA duplex, an event that can impede both replication and transcription [41–44]. DHX9 unwinds D- and R-loops approximately 5-7-fold more efficiently than corresponding DNA and RNA forks [37]. DNA and RNA-based G-quadruplexes containing 3′ single-stranded tails are also substrates for DHX9 activity (Figure 3). Under similar conditions, DHX9 unwinds RNA G-quadruplexes most efficiently, followed by R-loops, DNA G-quadruplexes, D-loops, RNA forks, and DNA forks, in descending order of helicase activity [37]. Another non-canonical structure is triplex DNA, which is composed of three DNA strands with the third strand bound to the duplex *via* Hoogsteen base pairing [45–47] (Figure 3). DHX9 is able to resolve triplex DNA structures, displacing the third strand with a 3′ to 5′ polarity, and displaying preference for triplexes having a 3′ single-stranded overhang on the third strand [2]. DHX9 has far greater helicase activity when presented with this substrate compared to blunt triplexes, triplexes with 5′-overhangs, duplexes (either with blunt ends or overhangs), and forked duplexes [2]. In sum, these data demonstrate that irrespective of the substrate, efficient DHX9 helicase activity requires a 3′ single-stranded tail, which may serve as an anchor for enzyme binding. In terms of substrate, it appears that DHX9 is a structure-specific helicase, with a higher propensity for unwinding RNA-containing substrates, and with a preference for more complex, multi-stranded nucleic acid structures.

The biochemical properties of DHX9 are similar to those of SV40 large T-antigen, a viral DNA/RNA helicase with a 3′ to 5′ polarity and low selectivity for dNTPs/rNTPs [48]. DHX9 shares many similarities with the RecQ helicases WRN and BLM, which play important roles in the maintenance of genomic stability. WRN and BLM are structure-specific helicases that unwind triplex DNA structures [49], D-loops, and R-loops, with the same 3′ to 5′ polarity as DHX9 [37, 50–53]. DHX9 and WRN both preferentially unwind DNA:RNA heteroduplexes with greater efficiency compared to DNA:DNA homoduplexes [35]. However, there are notable differences amongst these enzymes as well: whereas WRN and BLM can resolve Holliday junctions (another intermediate in homologous recombination), DHX9 cannot [37, 54, 55]. DHX9 can use both DNA- and RNA-based G-quadruplexes as substrates, while WRN and BLM can only resolve DNA G-quadruplexes [37, 56, 57]. DHX9 and the DHX36 helicase are the only enzymes thus far reported to unwind both DNA and RNA G-quadruplexes [58, 59].

Molecular cloning of the various domains and mutational analysis revealed that the two dsRBD domains at the N-terminus and the OB-fold and RGG-box at the C-terminus are dispensable for the NTPase and helicase activities of DHX9, and suggests that a minimal functional helicase likely consists of residues 313-1160 (Figure 1B) [25, 26]. The helicase-associated domain 2 (HA2) was found to be necessary for DHX9's unwinding activity [26]. A point mutation in Motif I (GCGKT to GCGRT) effectively abrogated ATP binding and ATPase activity, supporting sequence and structure data indicating this to be the site of NTP binding [60]. The two dsRBDs show specificity for binding to dsRNA, with optimal binding occurring when both dsRBDs are present. Although not required for unwinding, they enhance the catalytic activity by promoting binding of DHX9 to substrate RNA [26]. The RGG-box, on the other hand, binds specifically to ssDNA, with a lower affinity for ssRNA [25]. While the structure of full-length DHX9 has not been elucidated, it is thought that the N-terminal, helicase core, and C-terminal domains may be in close spatial proximity to each other and that the dsRBDs and RGG domains may help regulate or modulate the helicase activity [25]. For example, the dsRBDs and RGG domains may initiate binding to nucleic acids, and may cooperatively recognize single-stranded/double-stranded junctions. This binding may effect an allosteric change to activate the NTPase/helicase activity of DHX9, a situation which is observed in other dsRBD-containing helicases such as the double-stranded RNA-activated protein kinase DAI and the double-stranded RNA adenosine deaminase DRADA [61, 62].

## BIOLOGICAL CHARACTERIZATION OF DHX9

### Cellular localization of DHX9

Although DHX9 is predominantly a nuclear protein, it is able to shuttle to the cytoplasm to carry out some of its functions in translational regulation and miRNA processing [18]. It also migrates into the cytoplasm as a consequence of transcriptional inhibition and during mitosis [63]. During mitosis, release into the cytoplasm starts in prophase, which is marked by chromosomal condensation and breakdown of the nuclear envelope. Exclusion from the nucleus reaches a maximum during metaphase, where the condensed chromosomes align at the center of the cell, and DHX9 reenters the nucleus during telophase, where the nuclear envelope reforms [63, 64]. Shuttling is dependent on a nuclear localization signal (NLS) and a nuclear export signal (NES), both located at the C-terminal region (Figure 1B), and nuclear import is mediated by the classical importin-α/β dependent pathway [28, 65]. Nuclear translocation also requires methylation of arginine residues in the NLS by the protein arginine methyltransferase PRMT1 [66]. In addition, there is evidence that nuclear localization is mediated by the neuronal kinesin KIF1Bβ and its binding partner exportin-2 (XPO-2) [67]. The nuclear export pathway utilized by DHX9 remains to be elucidated, but it has been determined to be insensitive to leptomycin B, a drug that specifically blocks the CRM-1-dependent nuclear export pathway [65, 68, 69]. Aside from the NLS and NES region, subcellular localization may also depend on other functional domains. For example, a fragment of DHX9 containing dsRBDI and dsRBDII was found to localize to the cytoplasm, and mutation of the RNA PolII-binding MTAD resulted in nuclear localization defects [70].

Localization within the nucleus is dynamic and dependent on species, cell type, and context. In human cells, DHX9 is normally localized to the nucleoplasm and excluded from the nucleolus [29, 71]. However, under conditions of RNA PolII-mediated transcriptional inhibition, growth arrest, or stress induced by viral replication or low temperature treatment, DHX9 is translocated into the nucleolus [29, 71, 72]. Specifically, it localizes to the dense fibrillar components (DCFs) within the nucleoli, where ribosomal RNA biogenesis takes place [71]. Transport into the nucleolus is dependent on functional NTPase and helicase activity and is mediated by the dsRBDII and C-terminal nuclear transport domains [72]. In certain tumour cells, such as the breast cancer carcinoma line MCF-7, DHX9 is situated at the nucleolar periphery, bound to a F-actin network, and depolymerization of F-actin promotes entry into the nucleolus [73]. In murine cells, the opposite situation is observed: DHX9 is enriched in the DFCs of nucleoli but shuttles out into the nucleoplasm upon RNA polymerase I-mediated transcriptional inhibition, thermal stress, or cell cycle arrest [29]. The reasons underlying the different nuclear localizations of human and murine DHX9 are not entirely clear, but likely have to do with the fact that the latter has a much larger RGG-box at the C-terminus. It is possible that mouse cells may have higher rRNA transcriptional requirements, and the nucleolar localization of DHX9 may be an adaptation to accommodate this.

### DHX9 expression, half-life, and role in development

DHX9 is an abundant protein and is ubiquitously expressed. Expression is high in the skin, small and large intestines, stomach, pancreas, kidney, breast, skeletal muscle, bone marrow, and reproductive organs. The liver, spleen, lung, heart, smooth muscle, adipose tissues, and lymph nodes show a moderate level of expression [74–76]. DHX9 appears to be relatively stable, and its protein and mRNA half-lives have been measured in several cell lines. In murine NIH3T3 fibroblasts, its half-life was determined to be 81.1 hours at the protein level and 13.9 hours at the mRNA level [77]. In murine renal mpkCCD epithelial cells, its protein half-life is ~48 hours [78, 79]. DHX9's mRNA half-life is shorter in mouse embryonic stem cells, at 5.3 hours [80]. DHX9 participates in a variety of important biological processes and in many primary and transformed cell lines depletion leads to a reduction in cellular fitness [72, 81, 82]; however studies using a conditional shRNA mouse model have shown that reduced levels of DHX9 in adult mice is not deleterious at the organismal level [82]. On the other hand, DHX9 is essential during embryonic development. Homozygous DHX9 knockout mice are embryonic lethal, with marked apoptosis in embryonic ectodermal cells, suggesting a function for the helicase in the differentiation of the embryonic ectoderm [83]. The DHX9 homologue in *C. elegans*, RHA-1, is essential for germline transcriptional control and proliferation. Deletion of RHA-1 results in loss of lysine 9 methylation on histone H3, leading to defects in germline transcriptional silencing and consequently defective chromatin organization, reduced germ cell mitosis, and aberrant meiosis [22]. In *Drosophila*, Maleless (MLE) is responsible for X-chromosome dosage compensation, a process initiated in early embryogenesis which is critical to male development [20]. Homozygous mutations in MLE was found to be lethal for male zygotes [19]. In humans, DHX9 interacts with the LIM homeodomain transcription factor LMX1B, which is essential in mesodiencephalic dopaminergic neuron development [84]. DHX9 also interacts with the stem cell-specific RNA-binding protein L1TD1, which forms a complex with Lin28 to regulate translation of the stem cell factor Oct4, thus suggesting a role for DHX9 in human embryonic stem cell renewal [85]. Hence, the current evidence indicates that DHX9 is essential to development in a number of organisms.

## BIOLOGICAL FUNCTIONS OF DHX9

DHX9 has a large number of interacting partners, a reflection of the many biological processes it participates in. Table 1 summarizes the known proteins, nucleic acids, or sequence elements with which DHX9 interacts. This diversity in partners and targets is likely a consequence of DHX9's numerous functional domains and its promiscuity in terms of substrates. It participates at multiple levels of gene regulation and is a major player in many aspects of RNA biology. The following is an overview of its roles in DNA replication, transcriptional and translational regulation, RNA processing and transport, microRNA biogenesis, and maintenance of genomic stability. This discussion will focus on human DHX9, for which the largest body of research exists; however, where appropriate, other species will be discussed as well.

**Table I:** Protein and nucleic acid interacting partners of DHX9

Interacting partner	Biological process	DHX9 homologue(s) characterized	DHX9 domains/amino acid residues involved (if known)	Reference(s)
**Protein partners**				
Importin-α	Nuclear import of DHX9	Human	NLS (1155-1173)	[1]
PRMT1	Nuclear import of DHX9	Human	NLS (1155-1173)	[2]
WRN	Replication	Human	dsRBDII and RGG-box	[3]
PCNA	Replication	Human		[4, 5]
Topoisomerase IIα	Replication/transcription	Human		[6]
Ku86	Replication/DNA repair	Human		[7]
Myxoma virus protein M029	Viral replication	Human		[8]
Influenza A virus protein NS1	Viral replication/transcription	Human		[9]
γH2AX	DNA damage response	Human	Helicase core domain (313–952)	[10]
PML	DNA damage response	Human		[11, 12]
PHF1	DNA damage response	Human		[13]
RNA polymerase II	Transcription	Human, *Drosophila*, *C. elegans*	MTAD (331-380)	[14–17]
CBP/p300	Transcription	Human	dsRBDI+dsRBDII (1-250)	[14, 15, 17]
BRCA1	Transcription/miRNA biogenesis	Human	dsRBDII (230-325)	[18, 19]
EGFR	Transcription	Human	623-1270	[20]
p65	Transcription	Human	1-649	[21]
β-actin	Transcription	Human	RGG-box (1150–1270)	[22]
osterix	Transcription	Human		[23]
MEF1	Transcription	Human		[24]
Mineralocorticoid receptor	Transcription	Human	1-331	[25]
MBD2a	Transcription	Human		[26]
TonEBP	Transcription	Human	N-terminus (1-250) and C-terminus (1062–1270)	[27]
Zic2	Transcription	Human		[28]
NF110	Transcription	Human		[29]
LMX1B	Transcription	Human		[30]
UBC9	Transcription	Human	1-137	[31]
EWS-FLI1	Transcription	Human	630-1020	[32]
DDX5 (p68)	Transcription/miRNA biogenesis	Human		[19, 33]
DDX17 (p72)	Transcription/miRNA biogenesis	Human		[19, 33]
LARP6	Translation	Human		[34]
Lin28	Translation	Human	N-terminus (1-300) and RGG-box (1161–1270)	[35]
L1TD1	Translation	Human		[36]
TCP80	Translation	Human		[37, 38]
Dicer	miRNA biogenesis	Human	dsRBDI+dsRBDII	[39, 40]
TRBP	miRNA biogenesis	Human	dsRBDI+dsRBDII	[39, 40]
Ago-2	miRNA biogenesis	Human	dsRBDI+dsRBDII	[39, 40]
MBNL1	RNA processing	Human		[41]
SMN	RNA processing	Human	RGG-box	[42]
hnRNP C	RNA processing	Human		[43]
F-actin	RNA transport	Human	C-terminus	[43]
HAP95	Viral RNA transport	Human	C-terminus	[44–47]
Tip-associated protein (Tap)	Viral RNA transport	Human		[44–47]
Sam68	Viral RNA transport	Human		[48]
Rev viral protein	Viral RNA transport	Human		[48, 49]
KIF1Bβ	Apoptosis	Human		[50]
IPS-1	Host antiviral response	Human	Helicase core domain and C-terminus	[51]
				
**Nucleic acid partners/sequence elements**				
pINK4A promoter (CGGACCGCGTGCGCTG)	Transcription	Human	dsRBDI+dsRBDII (1-250)	[52]
MDR1 promoter (CAAT-like sequence)	Transcription	Human		[24]
miR-483p	Transcription	Human		[27]
Viral TAR RNA	Viral transcription	Human	dsRBDII (235–249)	[53]
roX2 RNA	Transcription	*Drosophila*		[54]
5′UTR PCE of mRNAs	Translation	Human	N-terminus (1-300)	[55–57]
Collagen 5′UTR stem-loop	Translation	Human		[34]
p53 5′UTR IRES	Translation	Human		[37, 38]
Viral CTE	Viral RNA transport	Human		[44–47]

### DNA replication and maintenance of genomic stability

The *in vitro* experiments illustrating DHX9's ability to unwind complex nucleic acid structures suggest a role in DNA replication and maintenance of genomic stability, since these are transient intermediates that form during replication, transcription, or recombination and need to be resolved. This is supported by several lines of evidence. First, DHX9 associates with numerous proteins involved in DNA replication and/or the DNA damage response. These include the breast cancer specific tumour suppressor protein BRCA1, which remodels chromatin, facilitates orderly homologous recombination, and ensures DNA replication fidelity [86]. The replication proteins PCNA (proliferating cell nuclear antigen) and topoisomerase IIα also bind DHX9 [87–89]. DHX9 interacts with Ku86, an essential player in NHEJ-mediated DNA repair which has also been implicated in promoting nascent DNA synthesis at origins of replication [90, 91]. DHX9 itself is associated with origins of replication and is necessary for efficient nascent DNA production. Suppression of DHX9 causes a blockage in DNA replication, an event that activates a p53 stress response resulting in growth arrest and senescence in human diploid fibroblasts [81]. This defines a role for DHX9 in DNA replication and normal cell cycle progression.

Mechanistic insight into DHX9's role in DNA replication is provided by studies investigating its relationship with WRN, a RecQ ATP-dependent helicase containing both 3′to 5′ helicase and 3′to 5′ exonuclease activity. WRN is involved in DNA replication, recombination, and repair, and defects in this helicase are a cause of Werner's syndrome, a rare autosomal recessive genetic disorder characterized by premature aging, increased genomic instability, and increased cancer susceptibility. In terms of substrate specificities, DHX9 shares many similarities with WRN (see Biochemical and Structural Characterization of DHX9, *In vitro* characterization of DHX9). These two helicases were found to interact, with the interaction sites mapping to the dsRBDII and RGG domains of DHX9 and the N-terminal exonuclease domain of WRN [92]. DHX9 enhances the exonuclease activity of WRN and stimulates the WRN-catalyzed unwinding of Okazaki fragment-like DNA:RNA hybrids and Holliday junction-like structures. Because DHX9 itself does not catalyze unwinding of either of these substrates, it is thought that it may aid in resolving RNA secondary structures at the 5′end of the Okazaki-like fragments [35, 92]. Okazaki fragments are formed on the lagging strand during DNA replication [93]. The *in vitro* results suggest that DHX9 may be loaded onto these sites, aiding WRN to remove the primer RNA-containing Okazaki fragments and promoting lagging strand maturation. Holliday junction-like intermediates are formed during replication fork stalling [94]. By resolving these structures and converting them to functional replication forks, DHX9 and WRN may act to ensure efficient DNA replication. Because both DHX9 and WRN interact with common replication-associated proteins such as PCNA and Ku86, it is possible that they may function as part of a larger replication complex [87, 88, 90, 95, 96].

DHX9's involvement in maintenance of genomic stability has been demonstrated both *in vitro* and *ex vivo*. *In vitro*, DHX9 binds to H-DNA, a naturally occurring intra-molecular DNA triplex [97]. H-DNA is an aberrant structure which induces genomic instabilities, such as gross rearrangements, point mutations and double-stranded breaks. Suppression of DHX9 in U2OS cells overexpressing an H-DNA-forming sequence derived from the human c-MYC gene promoter resulted in a significant increase in mutagenic events. Thus, DHX9 is purported to limit genomic instability by resolving H-DNA [97]. The ability of DHX9 to unwind G-quadruplexes is also suggestive of a role in genome maintenance. DNA G-quadruplexes are formed at G-rich telomeric sequences and may protect chromosomal ends against nucleases. However, these structures need to be resolved to permit telomere synthesis by the telomerase enzyme [98]. DHX9 may play a role in telomere maintenance by resolving telomeric G-quadruplexes, and this is supported by research showing that its interacting partner WRN plays a role in telomere processing [99]. Although DNA G-quadruplexes are most abundant at telomeres, they are also found at 90% of human DNA replication origins, which are GC-rich [100–102]. They can lead to replication fork stalling and represent an impediment to the replication process [103, 104]. The presence of DHX9 at origins of replication and its documented ability to cooperate with WRN in resolving stalled replication fork-like structures suggests that DHX9 may aid in promoting efficient replication by resolving G-quadruplexes.

Further evidence supporting a role in DNA repair and maintenance of genomic stability is demonstrated by evidence that DHX9 is phosphorylated by DNA-PK, a major player in NHEJ-mediated DNA repair. This is further corroborated by the observation that DHX9 interacts with Ku, the DNA binding subunit of DNA-PK [90]. DHX9 was found to interact with γH2AX *via* its helicase core domain, and this association is significantly increased upon actinomycin D treatment, where DHX9 accumulates in RNA-containing nuclear bodies adjacent to γH2AX foci [105]. Upon DNA damage, DHX9 also localizes to promyelocytic leukaemia (PML) nuclear bodies, which are involved in the DNA damage response [106, 107]. Association of DHX9 with sites of DNA damage is consistent with its interaction with the polycomb group protein PHF1, which is recruited to double-stranded breaks upon exposure to DNA damage [108].

### Transcriptional regulation

The *in vitro* experiments showcasing DHX9's ability to unwind RNA forks, R-loops, and RNA-based tetraplexes strongly suggested a role in transcription, since these are aberrant structures formed during stalled transcription. By resolving them, it was postulated that DHX9 may act to speed up transcriptional events. The earliest evidence of its role in transcription *in vivo* was studies in *Drosophila* that showed that MLE associates with the X chromosome and regulates dosage compensation. Although expressed in both males and females, MLE is bound to hundreds of sites along the X chromosome of males but not females [20]. The male X-chromosome shows a diffuse morphology, and male MLE homozygous null mutants show reduced transcription rates along the entire X chromosome and die during the larval stage [109]. Binding of MLE to the X-chromosome is RNA dependent, as it can be disrupted by RNase treatment [110], and it has been shown to interact directly with roX2, a non-coding RNA that forms part of the dosage compensation complex [111]. The NTPase and helicase activities of MLE are essential to its role in dosage compensation [34].

In mammals, dosage compensation occurs through an entirely different mechanism and there is no evidence that DHX9 plays a sex-specific role in mammalian development. Nevertheless, it has been implicated in transcription activation. DHX9 was discovered to be a bridging factor between the transcriptional co-activator CREB-binding protein (CBP)/p300 and RNA PolII. During CBP/p300-mediated transcriptional activation, phosphorylation of the cAMP response element-binding protein (CREB) results in complex formation with co-activator CBP/p300 and binding to a cAMP responsive element (CRE) in the promoter of specific genes [112]. DHX9 binds directly to both CBP/p300 and RNA polymerase II, recruiting the latter to the CREB/CBP/p300 complexes at the promoter [113]. Interaction with RNA PolII is mediated through the 50 amino-acid MTAD region which contains six hydrophobic residues conserved amongst DHX9 homologues [60, 114]. Mutational analyses indicate that three tryptophan residues within the MTAD are essential for PolII binding and transcriptional activation [60, 114]. Interaction with CBP occurs in a region between amino acids 1-250 in DHX9, where the two dsRBDs lie [113]. Aside from recruitment of PolII, the NTPase/helicase activity of DHX9 is also important for CREB-dependent transcription, suggesting a dual mechanism of transcriptional regulation [60]. The function of the MTAD is conserved in *Drosophila* and *C. elegans*. DHX9 from both species are able to recruit PolII and activate transcription via the MTAD [115].

Other instances of human DHX9 serving as a bridging factor between PolII and transcriptional co-factors have been uncovered. DHX9 links PolII to BRCA1, which in addition to its roles in DNA replication and DNA repair, also functions in transcriptional regulation [116]. Binding to BRCA1 occurs between a region in the N-terminus of DHX9 (residues 230-325) and the C-terminus of BRCA1, and resulted in transcriptional activation in reporter assays [117]. Although both located at the N-terminus, it is notable that the binding region for BRCA1 is distinct from that for CBP/p300 (residues 1-250). Nuclear β-actin is another component of the transcription pre-initiation complex [118], and DHX9 serves as an adaptor to link it with PolII. This interaction was reported to enhance transcription from the actin-dependent CSF-1 promoter. Contrary to what was observed in the case of CBP/p300, DHX9 interacts with β-actin *via* its C-terminal RGG region, and its catalytic activity is not required [119].

DHX9 participates in nuclear factor-κB (NF-κB)-mediated transcriptional activation, where members of the NF-κB family including p65, RelB and c-Rel upregulate transcription in response to exposure to a variety of inducers, such as interleukin-1 and tumour necrosis factor. DHX9 directly binds p65 and enhances NF-κB-dependent transcriptional activation, an event dependent on functional NTPase/helicase activity [120]. Since p65 is known to utilize CBP/p300 as a co-activator [121], it is possible that DHX9, p65, CBP/p300 and PolII may all be part of the same transactivation complex. Activation of CREB/CBP/p300-mediated transcription by the mineralocorticoid receptor [122], the methyl-CpG binding domain protein 2 (MBD2a) [123], and the E2-like enzyme UBC9 [124] are also dependent on interaction with DHX9. Other known DHX9-binding transcription activators include the osteoblast-specific transcriptional factor osterix [125], nuclear factor 110 (NF110) [126], the Zic2 zinc finger protein [127], topoisomerase IIα [89] and LMX1B [84].

DHX9 also binds directly to promoters in a sequence specific manner. It enhances transcription of the tumour suppressor p16INK4A by binding specifically to the sequence 5′CGGACCGCGTGCGC3′ within its promoter [128]. Another example of selective transcriptional regulation is that of the multidrug resistance gene 1 (MDR1). DHX9 is a component of the MDR1 promoter-enhancing factor (MEF1) complex and binds to the CAAT-like *cis*-acting element in the MDR1 promoter [129]. As well, DHX9 participates in EGF receptor (EGFR)-mediated transcriptional activation. Here, EGFR translocates from the cell surface to the nucleus in response to EGF signaling and activates transcription *via* an AT-rich sequence (ATRS) in the promoter of target genes [130]. EGFR-responsive genes include cyclin D1 and iNOS. EGFR lacks a DNA-binding domain; DHX9 mediates this interaction by simultaneously binding both EGFR and the promoter ATRS [131].

DHX9 can interact with RNA to regulate transcription. It has been found to bind both the insulin-like growth factor 2 (IGF2) mRNA and miR-483p, a microRNA that enhances IGF2 transcription, promoting the miR-483-5p-mediated induction of IGF2 mRNA [132]. In an example of regulation of viral nucleic acids, it interacts with the viral transactivation response element (TAR) RNA *via* its dsRBDII to stimulate HIV-1 transcription, a process that is dependent on DHX9's ATPase and PolII-binding activities [133, 134].

In addition to activating transcription, it appears that in some situations DHX9 can also repress it. Association with the transcriptional activator TonE (tonicity-responsive enhancer)-binding protein (TonEBP) inhibits TonEBP activity [135]. Although the mechanism is not clear, it is possible that DHX9 may recruit other proteins that directly inhibit TonEBP. The *C. elegans* homologue, RHA-1, enables normal germline proliferation and development by silencing transcription. A temperature-sensitive mutant, RHA-1(tm329), caused loss of lysine 9 histone H3 methylation (normally associated with silenced chromatin) and resulted in transcriptional desilencing. This resulted in defects in germ cell mitosis, meiosis, and gametogenesis, leading to a sterile phenotype [22]. Again, the mechanism of how RHA-1 silences transcription is unclear, but it may be direct (e.g. by promoting formation of heterochromatin) and/or indirect (e.g. by recruiting transcriptional repressors).

### Translational regulation

Beyond its role in transcription, DHX9 also participates in regulation of gene expression at the translational level. Many mRNAs contain highly structured 5′UTRs, a feature that represents an impediment to translational initiation, the rate-limiting step of cap-dependent translation. The eIF4A (DDX2) subunit of the eIF4F complex is responsible for unwinding structures to facilitate ribosome recruitment to the mRNA template [136–138]. However, a subset of mRNAs contains complex structural elements in their 5′UTRs that require resolution by additional specific helicases. One such element is the 5′post-transcription control element (PCE). Originally identified in the 5′ long terminal repeats of avian spleen necrosis virus [139] and subsequently in the 5′UTR of other retroviral RNAs, such as those found in HIV-1 and HTLV-1 (e.g. gag RNA), the PCE forms a complex secondary structure containing two stem-loop structures [140]. A PCE is also present in the 5′UTR of the cellular transcription factor JUND. DHX9 is necessary for the efficient translation of viral and JUND mRNA containing the PCE. It associates with structural features of the PCE *via* conserved lysine residues in the distal α-helices of the two dsRBDs and facilitates translation of said mRNAs by stimulating polyribosome incorporation [141, 142]. The NTPase/helicase activity of DHX9 is required for this function [143]. Association with PCE is sequence-specific and occurs both in the nucleus and in the cytoplasm, indicating it to be an early event in the post-transcriptional expression of PCE-containing mRNA and may satisfy a RNA-surveillance checkpoint that ensures efficient translation in the cytoplasm [141]. It has been proposed that DHX9 induces RNA-protein and RNA-RNA rearrangements to enable efficient association of ribosomes and thus increase the rate of protein synthesis. It is also possible that DHX9 stimulates ribosome recycling by securing circularization of the mRNA template *via* interaction with the poly(A)-binding protein (PABP), although this has not been verified [141]. Since JUND regulates cell growth in response to stress, its selective and tightly controlled translational regulation by DHX9 provides a means to rapidly link these two responses.

DHX9 also facilitates translation of type I collagen, another mRNA with a unique 5′UTR structural element. Type I collagen is a heterotrimer composed of two α1(I) polypeptides and one α2(I) polypeptide. The mRNAs encoding both polypeptides contain a unique 5′ stem-loop structure (5′sL) in their 5′UTR. La ribonucleoprotein domain family member 6 (LARP6) is known to bind with high affinity to the 5′sL [144]. DHX9 does not interact directly with the type I collagen 5′UTRs, but instead forms a complex with LARP6, which tethers it to the 5′sL. This enables polysome loading and efficient translation initiation. As is the case with PCE-mediated regulation, binding of DHX9/LARP6 to the 5′sL occurs in the nucleus as well as the cytoplasm, indicating that regulation begins prior to the onset of translation initiation [145]. In another example of regulation of a specific set of mRNAs by DHX9, the helicase interacts with Lin28 to enhance translation of Lin28 target mRNAs. Lin28 is a RNA-binding protein which plays a role in development, cell growth, pluripotency, and differentiation. It was first characterized as a key player in the biogenesis of let-7 family miRNAs [146]. More recently, it has been shown to regulate the translation of select mRNAs including IGF-2, the key pluripotency factor Oct4, histone H2a, cyclins A and B, and CDK4 [147–150]. DHX9 interacts with Lin28 *via* both its N- and C- terminal regions. This interaction promotes DHX9 association with polysomes and stimulates translation of Oct4 mRNA [148, 151]. It is thought that once recruited to Lin28 target mRNAs, DHX9 may aid in resolving inhibitory secondary structures. Further support for a role in Lin28-mediated translation regulation is provided by evidence that DHX9 also interacts with L1TD1, which forms a complex with Lin28 to regulate Oct4 translation in human embryonic stem cells [85].

Recent studies indicate that DHX9 also helps regulate IRES-mediated translation. Exposure to DNA damaging agents causes stabilization of the p53 tumour suppressor protein. However, recent evidence has shown that p53 translation is also increased upon DNA damage [152–154]. It was discovered that p53 mRNA contains an IRES in its 5′UTR [155, 156]. DHX9 was found to bind simultaneously to the p53 IRES and to translation control protein 80 (TCP80). DHX9 and TCP80 cooperatively stimulate p53 IRES-mediated translation. This stimulation is significantly enhanced upon exposure to DNA damage as a result of increased binding of TCP80 to the p53 IRES and improved interaction between DHX9 and TCP80. It is predicted that DHX9 likely helps unwind the p53 5′IRES, thereby promoting efficient translation [157, 158].

### MicroRNA biogenesis and processing

Involvement in microRNA (miRNA) biogenesis and processing is another means by which DHX9 regulates post-transcriptional gene expression. It associates with pri-miRNA, and along with its binding partner, BRCA1, forms part of the DROSHA microprocessor complex, which also contains DGCR8, DDX5, and DDX17. The concerted action of DHX9 and BRCA1 enhances processing of the pri-miRNA into their mature forms [159]. Further downstream in the miRNA processing cascade, DHX9 was also identified as a component of the active RISC. It binds directly to Dicer, TRBP, Ago-2, and siRNA duplexes, and association with all these components is mediated by key residues in dsRBDI and dsRBDII [160]. Isothermal titration calorimetric assays revealed that dsRBDI has a higher binding affinity than dsRBDII for the siRNA, but both dsRBDI and dsRBDII act cooperatively to bind the duplex [31]. The DHX9 ATPase/helicase activity is important but not absolutely essential for its association with Dicer, TRBP, and Ago-2, as an ATPase mutant only partially impaired these interactions. DHX9 depletion inhibits siRNA- and shRNA-mediated gene silencing in vivo and reduces siRNA association with the RISC, thus demonstrating that DHX9 is an essential component of the RISC and defining a role for it as a siRNA/miRNA loading factor [160].

### RNA processing and transport

As described above, human DHX9, while normally excluded from the nucleolus, can shuttle into said compartment under conditions of transcriptional inhibition or cellular stress [29, 71, 72]. DHX9 has also been identified in human prespliceosomes [161], binds to both mRNA and pre-mRNA [63], and interacts with the splice regulator muscleblind 1 (MBNL1) [162] as well as the survival motor neuron (SMN) protein, a component of small nuclear ribonucleoproteins (snRNPs) involved in pre-mRNA splicing [163–165]. This suggests a role in mRNA splicing and is supported by evidence that DHX9, in concert with ADAR2 (an adenosine deaminase that acts on RNA) coordinates the editing and splicing of glutamate receptor subunit B pre-mRNA. mRNA editing and splicing are competing events - ADAR2 editing requires a stable stem-loop, which may sequester the 5′ splice site. It is thought that DHX9 helps overcome this splicing inhibition by resolving the stem-loop [166]. An example of coordinated editing and splicing can also be found in *Drosophila*. In a process distinct from its role in dosage compensation, MLE links editing and splicing of the para sodium channel pre-mRNA. A mutation near the NTP-binding site of MLE results in aberrant splicing and exon skipping, again suggesting that the *Drosophila* DHX9 homologue may act to resolve secondary structures concealing splice sites [167, 168].

A role in RNA transport is suggested by the observation that DHX9 binds directly to both filamentous actin (F-actin) and hnRNP C1 in the nucleus, mediating their association [169]. F-actin is a major component of the nucleoskeleton, a network of interacting structural proteins that provides a scaffold for transcription, chromatin remodeling, and RNA processing and transport [170]. By facilitating the association of hnRNP C1, which plays a role in pre-mRNA processing, to F-actin, DHX9 enables efficient processing and transport of mRNAs. DHX9 also plays a role in the transport and splicing of viral RNAs. Nuclear export of simian type D retroviruses is dependent on host cellular proteins and mediated by a cis-acting constitutive transport element (CTE) on the viral RNA. DHX9 binds specifically to the CTE and interacts with two other shuttle proteins, Tip-associated protein (Tap) and HAP95, to allow export of CTE-containing viral RNAs [171–174]. DHX9 also facilitates transport of more complex viral RNAs such as HIV-1. Here, it cooperates with the viral protein Rev and cellular protein Sam68 to mediate nuclear export of viral RNAs containing the Rev response element (RRE) [175–178]. In addition, DHX9 modulates HIV-1 RNA splicing, a function that is mediated by its OB-fold [27].

## IMPLICATIONS OF DHX9 IN CANCER AND ITS TREATMENT

The role played by DHX9 in a multitude of cellular processes, including multiple levels of gene regulation, and its association with a large number of key regulatory binding partners, makes it an important protein in maintaining normal cellular homeostasis. However, this also means that defects in DHX9 may have serious effects on cell growth or viability, and usurpation of its functions may lead to a variety of human diseases.

### Implication of DHX9 in cancer

The human DHX9 gene maps to the major susceptibility locus for prostate cancer at chromosome band 1q25 [24], and DHX9 expression is under control of the transcription factor SOX4, which is overexpressed in prostate cancer [179]. Interaction of SOX4 with inhibitory protein plakoglobin inhibits binding of SOX4 to the DHX9 promoter and results in reduced DHX9 expression [180]. SOX4 also regulates transcription of RISC components Dicer and Ago-1, both of which interact with DHX9 [160, 179], and in fact, DICER itself is overexpressed in prostate cancer [181]. These studies suggest that DHX9 may play a role in prostate cancer development, although further work is needed to validate a direct mechanistic link. DHX9 is overexpressed in several cancer types. Two separate studies analyzing DHX9 levels in panels of lung cancer samples showed that DHX9 is overexpressed in tumour samples compared to normal lung tissues [182, 183], and that DHX9 overexpression was correlated with poorer patient survival [183].

As discussed previously, DHX9 interacts with EGFR to activate transcription of EGFR-responsive genes. EGFR is an oncogene overexpressed in several human cancers and drugs targeting EGFR are in clinical use (e.g. gefitinib, erlotinib, and cetuximab) [184–187]. An analysis of a panel of human breast tumour samples showed a strong positive correlation between the nuclear expression of DHX9, EGFR, and the EGFR target cyclin D1 [131]. Mutations in BRCA1, a DHX9-interacting tumour suppressor, increases risk of breast and ovarian cancers. Expression of a truncated form of DHX9 spanning the BRCA1 binding site (residues 89-344) but lacking other functional domains in normal mammary epithelial cells inhibited recruitment of BRCA1 to sites of DNA repair and resulted in pleomorphic nuclei, tetraploidy, and aberrant mitoses with extra chromosomes - a phenotype similar to that observed in BRCA1-deficient cells [188]. Furthermore, sequence analysis of DHX9 in a cohort of 96 breast cancer individuals from high-risk French Canadian families who do not harbour BRCA1/BRCA2 mutations identified two missense mutations (P89A and S625C) that lie in the dsRBDI (within the CBP/p300 binding site) and the helicase core domain, respectively [189]. The importance of these variations awaits further assessment.

DHX9 is also implicated in osteosarcoma and Wilms' tumour. Gene expression profiling of osteosarcoma cell lines showed overexpression of DHX9 in cells with high metastatic ability compared to those with low metastatic ability. Several other genes in the NF-κB pathway were upregulated as well; since DHX9 is a NF-kB binding partner, this supports a role for NF-κB signaling in osteosarcoma metastasis [190]. DHX9 cooperates with the miRNA miR-483-5p to induce IGF2 expression. Both miR-483-5p and IGF are overexpressed in Wilms' tumours and sarcomas, and ectopic expression of miR-483-5p in sarcoma cells and mouse xenographs enhances tumourigenesis [132].

Although much research implicates DHX9 as a promoter of tumourigenesis, there are also indications that it has tumour-suppressive properties. Its ability to unwind aberrant polynucleotide structures and to aid WRN in ensuring the fidelity and efficiency of DNA replication shows that it plays a role in maintaining genomic stability. It also activates transcription of p16INK4A, a tumour suppressor [128]. Its cooperation with the tumour suppressor BRCA1 in activating its target genes is also suggestive of an anti-tumour function [117]. It is a downstream mediator of KIF1Bβ tumour-suppressor function in neuroblastoma. KIF1Bβ-mediated activation of the pro-apoptotic XIAP-associated factor 1 (XAF1) and subsequent induction of apoptosis requires nuclear localization of DHX9 [67]. Perhaps the most compelling evidence is the IRES-mediated upregulation of p53 translation by DHX9 and TCP80 (see Biological Functions of DHX9, Translational regulation). It was found that cellular levels of DHX9 and TCP80 positively correlated with the efficiency of p53 IRES-mediated translation and effective induction of p53 signaling in response to DNA damage. Specifically, the breast cancer cell lines, ZR75-1 and MDA-MB-175, which express wild-type p53 but do not exhibit p53 induction following DNA damage, contain extremely low levels of both DHX9 and TCP80 compared to MCF-7 cells (which show a normal p53 response), and exhibit low p53-IRES activity when exposed to DNA damaging agents. IRES-mediated p53 translation was rescued by overexpression of DHX9 and TCP80 [158]. This shows that DHX9 levels can have a direct effect on the ability of p53 to suppress tumourigenesis. It appears that the relationship between DHX9 and oncogenesis is a complex one that may be dependent on cellular context and/or levels or activity of its interacting partners.

### DHX9 as a potential chemotherapeutic target

Recent research has supported the notion of targeting DHX9 as a chemotherapeutic approach. DHX9 was identified as a modifier of ABT-737 resistance in a mouse lymphoma model [191]. ABT-737 is a potent inhibitor of BCL-2, BCL-xL, and BCL-W pro-survival members, but only weakly binds MCL-1 and A1 [192, 193]. Using *Arf^−/−^*Eμ-*myc*/Bcl-2 mouse lymphoma cells, which overexpress MYC and exogenous BCL-2 and are resistant to ABT-737, a shRNA screen performed in search of sensitizers to ABT-737 uncovered DHX9 as a synthetic lethal hit [191]. DHX9 suppression overcomes resistance to ABT-737 by activating a p53 response through aggravation of replicative stress, resulting in upregulation of NOXA, inhibition of MCL-1, and ultimately apoptosis [191]. The *Arf^−/−^*Eμ-*myc*/Bcl-2 model recapitulates several clinically relevant features of non-Hodgkin's lymphomas; DHX9 may therefore show promise as a candidate target to suppress in combination with ABT-737 or its derivatives. Knockdown of DHX9 also synergizes with the glucocorticoid dexamethasone in human multiple myeloma cell lines [82, 194], suggesting a potential for targeting DHX9 in multiple myeloma as well.

Aside from the targeting of DHX9 in a combinatory approach, it has also effectively been targeted as a single agent. In a representative panel of human cell lines derived from different types of cancer, including multiple myeloma, osteosarcoma, breast, lung, and cervical cancers, suppression of DHX9 was lethal in the majority of these lines [82]. This was then modeled in a MYC-driven mouse lymphoma model, where DHX9 knockdown was found to be lethal both *ex vivo* and *in vivo* and extended survival of mice harbouring these tumours [82].

Ewing's sarcoma family tumours (ESFTs) is a pediatric cancer driven by a t(11;22) chromosomal translocation which fuses the 5′ transactivation domain of EWS with the 3′ ETS domain of the transcription factor FLI1. The resulting fusion protein, EWS-FLI1, is oncogenic and acts as a potent aberrant transcription factor [195]. DHX9 interacts with EWS-FLI1 and is required as a transcription co-activator of EWS-FLI1-responsive genes [196]. A small molecule (YK-4-279) blocking the interaction between DHX9 and EWS-FLI1 was found to induce apoptosis in ESFT cells and inhibit tumour growth in xenograph models [197]. YK-4-279 activity has been optimized through pharmacokinetic studies and an orally available formulation has been developed, thus making it a promising candidate for clinical development [198, 199].

In evaluating the feasibility of targeting DHX9 in as a neoplastic approach, two important considerations must be taken into account: (a) The availability of DHX9 inhibitors and (b) an achievable therapeutic index. At the moment, a specific inhibitor of DHX9 activity has not been identified - the approaches for curtailing DHX9 expression in the scenarios discussed above used shRNAs. However, attempts have been made to remedy this. A primary screening assay was recently developed to uncover inhibitors of DHX9 activity, where it was found that aurintricarboxylic acid prevents DHX9-mediated ATP hydrolysis [36]. Although this compound is promiscuous and therefore not an ideal DHX9 inhibitor, this screening approach can be implemented on a large-scale basis to search for more selective inhibitors. With regards to the therapeutic index, it has been demonstrated that whereas loss of DHX9 is lethal in various human and mouse cancer cells, prolonged systemic DHX9 suppression in a conditional shDHX9 mouse model is well tolerated at the organismal level. Mice harbouring reduced DHX9 levels exhibit no deleterious physiological or biochemical effects compared to control shRNA mice - surprisingly, even in highly proliferative tissues such as skin or intestines [82]. Hence, DHX9 suppression is tolerated in normal tissues and suggests that a therapeutic index can be achieved. The differential effect of DHX9 suppression in tumour cells *versus* normal tissues may be due to a higher dependency of the former on DHX9. Replication, transcription, translation, and many other biological processes are often deregulated in cancer, and since DHX9 is an important player in all of these processes, cancer cells may become “addicted” to DHX9. It should be noted that the shRNA mouse model used in the aforementioned study represents partial inhibition of DHX9. Because of the importance of DHX9 in many regulatory processes, it is quite possible that total loss of DHX9 (e.g. as in a conditional knock-out model) may be detrimental to the adult organism. A straight knock-out mouse model showed that DHX9 is embryonic lethal, but does not inform on the consequences of acute DHX9 loss in the adult [83]. If DHX9 were to be used as a chemotherapeutic target, careful dosing of DHX9 inhibitors would be essential in ensuring a good therapeutic index.

## IMPLICATIONS OF DHX9 IN OTHER DISEASES AND AGING

### Role of DHX9 in viral infection

Viruses can hijack various aspects of the host cell machinery for their own purposes. This includes using host proteins to facilitate the replication, transcription, translation, or transport of their own nucleic acids or proteins. DHX9 interacts with the viral TAR RNA to stimulate HIV-1 transcription [133, 134]. As indicated above, it facilitates translation of viral RNAs containing a PCE in the 5′UTR [141, 142] and mediates nuclear export of both CTE-containing (e.g. simian type D retrovirus) and RRE-containing (e.g. HIV-1) RNA [171–178]. DHX9's involvement in aiding many aspects of viral function serves to enhance infection efficiency, and indeed it has been implicated in promoting infectivity of a whole range of viruses including HIV-1 [27, 133, 134, 143, 175, 177, 200, 201], HCV [202] cytomegalovirus [203], adenovirus [204], and Hepatitis E [205], influenza A [206], myxoma [207], classical swine fever [208], and foot and mouth disease viruses [209].

Surprisingly, DHX9 has also been implicated in antiviral immune responses. For example, it is recruited to PML bodies in response to IFN-β stimulation and is phosphorylated by the dsRNA-binding kinase PKR [107, 133]. It also interacts with IFN-β promoter stimulator (IPS)-1 and acts as a sensor for double-stranded RNA to promote IFN and inflammatory responses [210]. The importance of DHX9 in innate immunity is highlighted by the observation that the DHX9 homologue is missing from chickens and ducks, thus rendering these species more susceptible to many viruses than mammals (e.g. the avian influenza H5N1) [211]. Avian influenza is particularly pathogenic in chickens because they are also deficient in another viral sensor, RIG-1 (DDX58), which is present in ducks and can partially compensate for the loss of DHX9 [211, 212]. The opposing roles of DHX9 in both pro-viral and anti-viral response suggests that there may be a tug-of-war between the host cell's attempts to combat viral infection and the viruses' attempts to hijack cellular machinery - a battle in which DHX9 appears to play a crucial part.

### Role of DHX9 in aging

DHX9 knockdown induces a pronounced p53-dependent growth arrest and premature senescence in primary human fibroblasts [81]. This is accompanied by genome-wide downregulation of genes involved in DNA replication, mitosis, and cell cycle progression, with the gene expression signature closely resembling that of replicative senescent cells. The phenotype is caused by an inhibition of DNA replication, which activates a p53-dependent stress response and results in upregulation of p21 [81]. This implies that defects in DHX9 may lead to accelerated aging, in a similar manner as the WRN and BLM helicases. Defects in WRN and BLM, which resemble DHX9 in their substrate specificities, lead to rare autosomal recessive disorders characterized by premature aging, growth retardation, increased genome instability, and increased cancer susceptibility.

### Role of DHX9 in autoimmune disease

Systemic lupus erythematosus (SLE) is an autoimmune disease in which the body launches an immune response against healthy tissues. It is characterized by the generation of antibodies against the body's own proteins, termed autoantigens, most of which are nuclear proteins. This leads to inflammation and complications such as skin rashes, photosensitivity, and atherosclerosis [213]. DHX9 was detected as an autoantigen in the sera of SLE patients [214]. Autoantibodies against DHX9 were found in ~6% of patients with SLE and this percentage was increased to 23% in SLE patients of Mexican descent, consistent with population differences in the manifestation of SLE. It is also more common in the early stages of disease [215, 216]. DHX9 is a substrate of caspase-3 cleavage during apoptosis and it is thought that the cleaved fragments produced may trigger an autoimmune response [214]. Hence, DHX9 may be clinically useful as a marker in aiding the diagnosis of SLE. Further work is needed to determine why DHX9 autoantibodies are generated in some SLE patients but not others, and whether there are additional subsets of SLE patients with particularly high instances of DHX9 as an autoantigen.

## CONCLUDING REMARKS AND FUTURE PERSPECTIVES

DHX9's interaction with an extensive and varied array of nuclear and cytoplasmic protein and nucleic acid partners indicates that it holds a privileged position as a central regulator of gene expression. Although much has been uncovered about DHX9 in the past three decades, there is still a lot of information to be gained with respect to its structure, substrates, binding partners, and functions. Future work should focus on obtaining greater mechanistic insight on DHX9 - for example, elucidating the processivity of its helicase activity, why it prefers certain substrates to others, and how it is able to recognize and bind to specific sequences or structures on DNA or RNA. As well, further characterization of non-human homologues would be of interest to determine if many of the functions and binding partners observed in humans are conserved across different species, or if there may be yet-undiscovered species-specific activities. Finally, the potential of using DHX9 as an anti-neoplastic or anti-viral target is still in its infancy. Additional studies characterizing the mechanistic consequences of DHX9 overexpression or mutation in various cancers, as well as studies in animal models of disease, would be beneficial in understanding the role of DHX9 in tumourigenesis, and the identification of DHX9 inhibitors would contribute to the feasibility of targeting DHX9 in chemotherapy.
